# Response of Patient-Derived Non-Small Cell Lung Cancer Xenografts to Classical and Targeted Therapies Is Not Related to Multidrug Resistance Markers

**DOI:** 10.1155/2009/814140

**Published:** 2009-06-14

**Authors:** Jana Rolff, Cornelia Dorn, Johannes Merk, Iduna Fichtner

**Affiliations:** ^1^Experimental Pharmacology & Oncology GmbH, Robert-Rössle-Street 10, 13122 Berlin, Germany; ^2^Experimental Pharmacology, Max-Delbrück-Center for Molecular Medicine, Robert-Rössle-Street 10, 13125 Berlin, Germany; ^3^Evangelische Lungenklinik, Lindenberger Weg 27, 13125 Berlin, Germany

## Abstract

Tumor cells that are nonsensitive to anticancer drugs frequently have a multidrug resistant (MDR) phenotype. Many studies with cell lines and patient material have been done to investigate the impact of different resistance markers at protein and mRNA level in drug resistance but with contradictory outcome. In the present study, 26 well-characterised patient-derived non-small cell lung cancer xenografts were used. The known chemosensitivity to etoposide, carboplatin, gemcitabine, paclitaxel and erlotinib was compared to the protein and mRNA expression of BCRP, LRP, MDR1, and MRP1. Further, four of these xenografts were short-term treated to analyse possible regulation mechanisms after therapeutic interventions. We found a borderline correlation between the *bcrp* mRNA expression and the response of xenografts to etoposide. All other constitutive mRNA and protein expression levels were not correlated to any drug response and were not significantly influenced by a short term treatment. The present results indicate that the expression levels of MDR proteins and mRNA investigated do not play an important role in the chemoresistance of NSCLC in the in vivo situation.

## 1. Introduction

 Lung cancer is still one of the most frequent cancers with about 1 million incidences worldwide each year. The 5-year survival rate is low with 10–15% compared to other cancers. For chemotherapeutic treatment the classical drugs like etoposide, gemcitabine, carbo- or cisplatin, vinorelbine, docetaxel, and paclitaxel are used. For some years also targeted therapies like tyrosine kinase inhibitors, like gefitinib, and erlotinib have been introduced into clinical trials. However, some patients seem to exhibit an intrinsic resistance or develop an acquired resistance under treatment. It was shown that active drug efflux transporters of the ATP binding cassette (ABC) were involved that actively extrudes a range of structurally and functionally diverse drugs [1, 2]. Three human ABC transporters are primarily associated with the multidrug resistance, namely, P-glycoprotein (P-gp, MDR1, ABCB1), multidrug resistance protein 1 (MRP1, ABCC1), and breast cancer resistance protein (BCRP, ABCG2). They have broad and, to a certain extent, overlapping substrate specificities and are involved in transport processes for a variety of drugs used in chemotherapy. So it was shown that etoposide can be transported by MRP1 [3] and MDR1 that is also able to cause resistance to bulky amphipathic drugs, such as paclitaxel [1]. The lung cancer related protein (LRP) is associated with multidrug resistance because it was found to be overexpressed in an NSCLC cell line selected for doxorubicin resistance that did not express MDR1 [4]. Moreover, it was reported that erlotinib was a substrate for BCRP [5–7].

Most studies used only small numbers of lung cancer cell lines selected for resistance or patient material that was correlated with clinical features [8, 9]. It was turned out that MRP1 played a major role in the intrinsic resistance. Further on, an activation of MDR1 expression during chemotherapy was suggested [10]. Additionally, it was shown that the response to Taxol-based chemotherapy was related to MDR1 but not LRP expression [11]. These partially conflicting data require further research.

Therefore we initiated a study in patient-derived NSCLC xenografts that were not selected for resistance and revealed a high coincidence with the original tumor [12]. We wanted to address the question if the level of resistance markers on mRNA or protein level is correlated with the response of xenografts to classical cytotoxic drugs (etoposide, carboplatin, gemcitabine, and paclitaxel) or targeted therapy (erlotinib).

## 2. Methods

### 2.1. Animal Experiments

26 recently established NSCLC xenografts were used for this study (Table 1). The chemosensitivity was tested recently [12] so here described only shortly.

All animal experiments were done in accordance with the United Kingdom Co-ordinating Committee on Cancer Research regulations for the Welfare of Animals and of the German Animal Protection Law and approved by the local responsible authorities. The chemotherapeutic responsiveness of the passagable tumors was determined in male NMRI:nu/nu mice. One tumor fragment each was transplanted subcutaneously to the mice. At palpable tumor size (50–100 mm*³*) mice each was randomised to treatment and control groups The following drugs and treatment modalities were used: etoposide (Vepesid, Bristol-Meyers Squibb) 10 mg/kg/d, qd 1–5, i.p.; carboplatin (Mayne Pharma Deutschland GmbH) 75 mg/kg/d, qd 1 and 8, i.p.; gemcitabine (Gemzar, Lilly Deutschland) 60–80 mg/kg/d, qd 1, 4, 7, 10, i.p.; paclitaxel (Taxol, Sigma) 12.5 mg/kg/d, qd 1–5, i.v.; erlotinib (Tarceva, Hoffmann-LaRoche) 50 mg/kg/d, qd 1–5, 8–12, orally. Doses and schedules were chosen according to previous experience in animal experiments and represent the maximum tolerated or efficient doses. The injection volume was 0.2 mL/20 g body weight. 

In this study, the four models 7406, 7433, 7700, and 7747 were selected because of their differential chemosensitivity (Table 1). 7406 was choosen because it was the only model that not responded to carboplatin and gemcitabine at once but responded to erlotinib. The other models were randomly selected but should represent the high response rates of all tumors to carboplatin and paclitaxel (models 7433, 7747) and gemcitabine and paclitaxel (7700). At the same time they should not respond to more than two drugs to keep the factors of influence low. For the short-term treated xenografts three mice each were randomised to treatment and control groups. The drug doses and application mode were the same as described above except that the treatment was carried out for three days. 24 hours after the last treatment the mice were sacrificed, tumors were snap frozen and stored at −80°C. Total RNA and protein were isolated for the analysis.

### 2.2. Real-Time PCR

RNA was isolated with RNA Isolation Kit (Qiagen) according to the manufacturers instructions. Total RNA was reversely transcribed using TaqMan Reverse Transcription Reagents (Applied Biosystems (AB)) and TaqMan quantitative real-time PCR performed using cDNA corresponding to 40 ng RNA per reaction. Gene and species specific primers for *bcrp, lrp, mdr1, mrp1,* and *β-actin* and TaqMan Fast Mastermix (AB) were used according to the manufacturers instructions and amplifications carried out on the StepOne Plus Real-Time PCR system (AB) with 45 cycles. Each sample was done in two replicates. Normalised ΔC_T_ values were obtained by subtracting the *β-actin* C_T_ from the gene of interest C_T_. Tumor samples have been done 2-fold and as positive controls MDA-MB-231/BCRP, A549 and MT3/ADR were used.

### 2.3. Immunoblotting

Lysates for immunoblotting were prepared by adding lysis buffer (150 mM NaCl, 20 mM Tris, 1% Triton X-100, 0, 5% sodiumdesoxycholate, 0.5% SDS, 2 mM EDTA, 2.5 mM sodium pyrophosphate, 1 mM *β*-glycerophosphat; pH 7.7) containing protease and phosphatase inhibitors (Sigma-Aldrich) to the tumor tissue. The protein concentration was determined using BioRad Protein Assay (BioRad Laboratories GmbH). Tumor lysates (20 *μ*g) were separated on 8% SDS-page polyacrylamide gels and transferred to nitrocellulose membranes. Membranes were blocked and incubated with the primary antibodies (BCRP, 801-029-C125, Alexis; LRP, 610512, BD) overnight at 4°C. The secondary antibody (115-035-003, Jackson Immuno Research) was conjugated with horseradish peroxidase. Protein bands were visualized using the enhanced chemiluminescence detection system (GE Health Amersham Life Science Inc). To verify equal protein loading, the blots were stripped and reprobed for *β*-actin (Sigma). MDA-MB-231/BCRP and A549 were used as positive controls.

### 2.4. FACS Analyses

One piece of each tumour was crudely cut into smaller pieces and further separated with a cell strainer till a cell suspension was obtained. Approximately 1 × 10^6^ cells were used for analyses. After blocking with goat serum, cells were incubated with the primary antibody (MDR1 557001, BD; MRP1, 557594, BD) and secondary Cy3-conjugated goat anti mouse antibody (115-165-146, Jackson Immuno Research). As positive control MT3/ADR breast cancer cells were used.

### 2.5. Statistical Analyses

Analyses of the mRNA or protein expression levels in comparison with the response to treatment have been done. The correlation according to Spearman and the *P*-values was calculated with the SPSS software. The correlation coefficient (*r*) could range between 0 (no correlation) and 1 (strong correlation).

## 3. Results

### 3.1. Constitutive Protein Expression in the 26 NSCLC Models

BCRP protein was detected in 18/26 xenografts with a weak to strong intensity. LRP could be found in 24 xenografts with different expression levels. Two NSCLC 7462 and 7668 lacked expression of LRP. MDR1 and MRP1 proteins were detected in all xenografts with an almost equal expression level (see Table 1).

### 3.2. Constitutive mRNA Expression in the 26 NSCLC Models

*Bcrp* was expressed in the xenografts in a ΔC_T_ range between 4 (7126) and 16 (7558). *Mdr1* was detected in all xenografts except in five (7166, 7187, 7336, 7343, and 7414). The highest expression with a ΔC_T_ value of 9 was found in xenograft 7462, the lowest level with a ΔC_T_ 22 in 7466. Nearly half of the other xenografts (13) had ΔC_T_ value in a dose range between 15 and 19. The expression of *mrp1* varied from ΔC_T_ values of 2 to 8. In 13 xenografts a ΔC_T_ value between 6 and 7 was found. The *lrp* levels ranged in all xenografts between ΔC_T_ 5 and 8, hence presenting a relatively homogeneous expression. *Bcrp* and *mdr1* had the most heterogeneous mRNA expression pattern, and the overall expression level of * lrp* and *mrp1* was higher than that of *bcrp* and *mdr1* in the 26 xenografts. 

A borderline correlation between chemosensitivity and mRNA expression was found in the comparison of etoposide and *bcrp* (*r* = 0.490). All 6 xenografts sensitive to etoposide showed a lower *bcrp* expression ΔC_T_ 13,8 (±1.6) whereas the resistant tumors had a mean ΔC_T_ of 9,6 (±3.3). The comparison of the *lrp, mdr1,* and *mrp1* expression with the chemosensitivity towards the different drugs revealed no further correlations.

### 3.3. mRNA Expression in the Xenografts after Short-Term Treatment

RNA was isolated after short-term treatment of the xenografts 7406, 7433, 7700, and 7747. 

**Figure 1:**
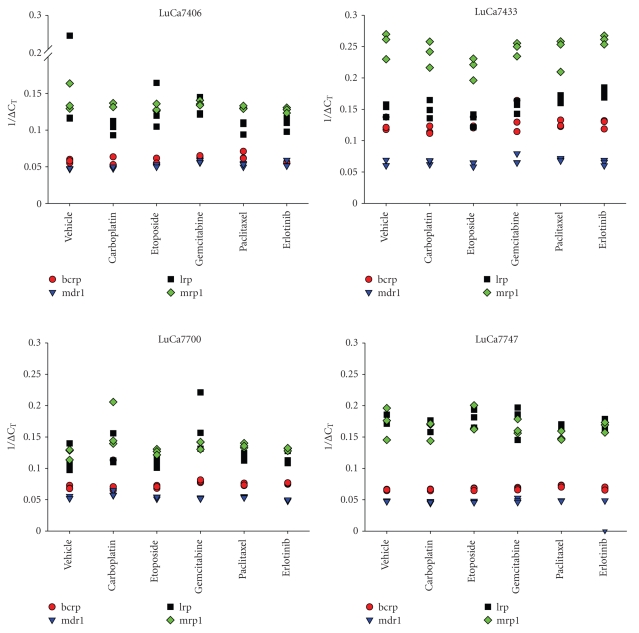
mRNA expression of *bcrp, lrp, mdr1,* and *mrp1* after short-term treatment in xenografts 7406, 7433, 7700, and 7747. Treatment was performed for three consecutive days. Three tumor samples per group were taken 24 hours after last treatment.

In all four xenografts the mRNA of *bcrp, lrp, mdr1,* and *mrp1* could be detected. For one and the same xenograft the mRNA expression was independent of the treatment (Figure 1). The ΔC_T_ values differed in a range of two. No significant up- or down-regulations of the mRNA after treatment with etoposide, carboplatin, gemcitabine, paclitaxel, and erlotinib could be observed.

### 3.4. Protein Expression in the Xenografts after Short-Term Treatment

The BCRP and MRP1 proteins were detected at a medium or weak expression level, whereas LRP had a strong expression in the all xenografts. MDR1 protein could be found in all xenografts. All groups of one model showed an equal expression level of BCRP, LRP, and MDR. There was no regulation detectable after treatment in each NSCLC xenograft (data not shown).

## 4. Discussion

A large number of studies dealing with questions of intrinsic or acquired drug resistance used cell line-based approaches. Hence, it was shown that amplification and overexpression of *BCRP* emerged as the dominant resistance mechanism in MDR1 and MRP1-deficient mouse fibroblast and kidney cell lines that were selected for resistance to etoposide [13]. In the present study, comparing etoposide response to *bcrp* mRNA expression the tendency was shown that all sensitive xenografts had a lower expression level than the nonsensitive tumors. Similar correlations could not be found at the protein level. Recently, it was demonstrated that erlotinib was a substrate for BCRP and MDR1 which may explain the resistance seen in the clinics [6]. In our study, we did not observe any correlation between response and BCRP or MDR1 expression neither at protein nor at mRNA level.

Various studies showed that the expression of *LRP* closely reflected the chemoresistance profile of many tumor cell lines and clinical cancer [4, 14–17]. Elevated LRP levels were observed in cell lines resistant to cytotoxic agents like doxorubicin, etoposide, vincristine, and cisplatin [4, 18, 19]. In nonselected NSCLC cells LRP protein and mRNA expression levels correlated with resistance to cisplatin [20]. However, in the present study, no correlation was observed regarding resistance to etoposide, carboplatin or other drugs and the expression of LRP. In other studies, likewise, no correlation with relevant clinical or clinicopathological parameters could be demonstrated [21, 22]. Anyway, in non-small cell and small cell lung cancer patients, the expression was different with the highest expression found in chemoresistant NSCLC [21].

In the present study, a relation of *MRP1* expression neither to cisplatin nor to etoposide response was seen. In contrast, other authors reported that MRP1 expression was correlated with lower chemosensitivity to etoposide, but not to cisplatin in lung cancer cell lines and patients [8, 23, 24]. NSCLC patients were found to exhibit mostly low, but occasionally high MRP1 mRNA expression levels [25]. Another study indicated that either one, or both, MDR1 or MRP1 was frequently expressed in NSCLC, and expression of *mrp1* was found to be predominant over *mdr1* at the mRNA level [26]. This could be confirmed in the present study as it detected almost equal mRNA expression levels among the xenografts. In general, the *mrp1* level was higher than that of the *mdr1*. 

For *MDR1* expression also contradictory literature exists. Some concluded that Taxol-based chemotherapy response of NSCLC patients was related to MDR1 but not LRP expression [11] while others suggested that MDR proteins (LRP, MDR1, and MRP1) may not play an important role in the chemoresistance and drug efflux of NSCLC cells [9]. We were not able to demonstrate any correlation between the chemosensitivity and the expression of MDR. Even after short-term treatment no remarkable changes of mRNA or protein could be observed.

One reason for the different results described in literature and found by us could be the model system used. While we used patient-derived xenografts that were not selected for any drug resistance, many other studies included cell lines passaged over years or selected for resistance under high drug concentrations. The *in vivo* situation is different because the drug availability and exact “in-tumor” concentration are not exactly known. However, the response rates of xenografts were similar to those observed in human Phase II studies with the same agents [12, 27]. Patient-derived xenografts allow the detailed investigation of therapy related markers and their dynamic regulation in a well-standardized and clinically related way.

Moreover, the multidrug resistance is regarded to be a multifactorial phenomenon in which more than the markers studied in the present study could be involved.

## Figures and Tables

**Table 1:** Chemosensitivity testing, constitutive protein, and mRNA expression of BCRP, LRP, MDR1, and MRP1 in 26 xenografts and the positive controls. Response: − negative: 100–50% T/C, + 35–50% T/C, ++ 21–35% T/C, +++ 6–20%T/C, ++++ 0–5% T/C, tox-toxic, n.t.—not tested; protein expression: − not detected, + weak, ++ medium, +++ strong expression; % of positive cells; mRNA expression: normalised ΔC_T_ values; etp—etoposide, carpl—carboplatin, gem—gemcitabine, paltx—paclitaxel, erlo—erlotinib.

						Before treatment							
	Chemosensitivity					Protein expression				mRNA expression			
LuCa	etp	Carpl	gem	paltx	erlo	BCRP	LRP	MDR1 [%]	MRP1 [%]	bcrp	lrp	mdr1	mrp1
7064	++	−	−	++	++	−	+	15.4	13.8	12.19	8.01	17.54	7.10
7126	−	−	+++	−	++	+++	+	15.3	18.2	4.17	5.04	18.18	3.45
7166	++	++	+	−	−	−	+++	13.7	13.8	15.46	6.45	0	7.16
7177	−	++	+++	−	++	−	++	14.5	29.5	10.42	5.33	16.97	2.02
7187	−	++	+++	−	−	−	++	15.4	18.3	8.13	6.90	0	7.55
7198	−	+	+	+	−	+++	++	27.2	20.9	10.13	6.18	21.24	6.25
7298	+	+	++	++	−	−	++	14.4	13.1	15.03	5.79	17.19	6.33
7336	−	(+)	++	+++	−	+++	+	13.3	13.6	8.08	6.68	0	7.02
7343	−	+++	+++	++	−	+	+	20.8	24.1	8.34	5.85	0	5.23
7387	−	−	+++	++++	−	++	++	15.4	29.9	6.15	7.64	10.90	7.57
7406	+	+	+++	+++	−	+++	+	29.4	22.4	14.87	7.61	14.97	8.10
7414	−	++	+++	+++	+	−	++	17.9	15.6	7.13	5.69	0	6.21
7433	−	+++	−	++++	−	−	+++	18.8	22.1	6.99	5.68	17.20	3.73
7462	−	+	++++	+++	++	+	−	39.8	31.3	7.65	5.24	9.36	7.78
7466	−	−	++++	++++	++	+	++	15.5	18.6	8.62	7.56	22.43	8.65
7506	−	++++	(+)	+++	−	+	+	65.8	19.0	10.96	7.07	15.94	6.98
7530	++++	−	tox	+++	−	+++	+	20.1	16.1	14.27	8.24	14.35	8.01
7558	−	++++	+++	−	−	+++	++	24.0	25.3	15.90	6.26	18.64	6.38
7612	−	++++	−	+++	−	−	+	17.7	15.5	11.09	5.89	16.98	6.04
7668	−	+++	tox	++++	−	+++	−	17.2	24.8	7.55	6.12	13.42	6.17
7700	−	−	++++	++	−	++	+++	18.1	13.9	15.81	6.01	17.32	7.96
7747	−	++	−	++	−	+	++	62.0	15.0	15.19	4.82	18.29	6.26
7766	−	+++	+	++	−	+	+	33.1	18.0	6.72	5.18	17.88	4.14
7860	+	−	++	+++	−	+	++	20.6	22.4	11.16	4.96	16.41	5.47
7913	−	+	(+)	+++	−	+	++	14.0	n.t.	13.50	5.64	18.47	6.17
7915	n.t	n.t	n.t	n.t	−	+	++	16.0	13.8	14.27	8.49	16.21	6.87

MDA-MB-231/BCRP										2.51			
A549											9.97		5.54
MT3/ADR												3.49	
